# Taxonomic revision of the Elephant Pupinid snail genus *Pollicaria* Gould, 1856 (Prosobranchia, Pupinidae)

**DOI:** 10.3897/zookeys.287.4617

**Published:** 2013-04-11

**Authors:** Bangon Kongim, Chirasak Sutcharit, Fred Naggs, Somsak Panha

**Affiliations:** 1 Department of Biology, Faculty of Science, Mahasarakham University, Kantharawichai, Maha Sarakham 44150, Thailand; 2 Animal Systematics Research Unit, Department of Biology, Faculty of Science, Chulalongkorn University, Bangkok 10330, Thailand; 3 Department of Life Sciences, The Natural History Museum, London SW7 5BD, United Kingdom

**Keywords:** Systematics, Indochina, Gastropoda, land snail, Southeast Asia, anatomy

## Abstract

The status of species currently assigned to the Southeast Asian Elephant Pupinid snail genus *Pollicaria* Gould, 1856 is reassessed. Shell, radular and reproductive morphology are investigated and analysed with reference to karyotype patterns previously reported and to distribution patterns among the species. Six previously described species are recognised: *Pollicaria gravida* (Benson, 1856), *Pollicaria myersii* (Haines, 1855), *Pollicaria mouhoti* (Pfeiffer, 1862), *Pollicaria elephas* (Morgan, 1885), *Pollicaria crossei* (Dautzenberg & d’Hamonville, 1887) and *Pollicaria rochebruni* (Mabille, 1887). A new subspecies, *Pollicaria mouhoti monochroma* **ssp. n.**,is proposed and a dichotomous key to species is provided.

## Introduction

Land operculate snails of the family Pupinidae generally possess a pupoid shell shape and exhibit a wide range of shell height from 5–50 mm. Apart from size, their often distinctive shells can also be distinguished from other members of the Cyclophoroidea by unique features of the genitalia, notably the long bursa copulatrix (Wenz 1938, Tielecke 1940). About 20 extant genera range from South Asia, East Asia to Southeast Asia, Melanesia, Micronesia and part of Australia (Solem 1959, Vaught 1989, Stanisic 1998, Stanisic et al. 2010). Fossil representatives are known from the European Cretaceous (Naggs and Raheem 2005) and British Eocene (Sandberger 1873). They generally occur in tropical forest and most commonly and abundantly in limestone areas. Fourteen pupinid genera have been recorded from Indochina (Kobelt 1902), including the very distinctive Elephant Pupinid genus *Pollicaria* Gould, 1856 which is endemic to the region.

Hitherto, nine nominal species of the *Pollicaria* have been described (Crosse 1885, Kobelt 1902, Gude 1921, Pain 1974). *Pollicaria*, as “*Hybocystis*”, was first revised by Crosse (1885) and by Fischer (1885) who detailed the anatomy. Crosse (1885) recognized four species of *Pollicaria* and separated those species into two species groups, which are now unrecognized. Subsequently, two additional species were described from Vietnam (Dautzenberg and ďHammonville 1887, Mabille 1887a). These six nominal species were revised by Kobelt (1902) and more recently Pain (1974). Relying solely on shell morphology, Kobelt (1902) placed *Pollicaria crossei* into synonymy with *Pollicaria rochebruni*. Pain (1974), partly followed Kobelt’s classification but recognized only three species: *Pollicaria gravida* (Benson, 1856), *Pollicaria myersii* (Haines, 1855) and *Pollicaria elephas* (Morgan, 1885), placing *Pollicaria mouhoti* into synonymy with *Pollicaria myersii*. However, Pain’s study was of limited value because it was based on an examination of few specimens and populations and did not examine the type specimens. Hence the true status of species still remains unresolved. Apart from the studies of Crosse (1885) and Fischer (1885) none of the subsequent studies on *Pollicaria* have used anatomical data or studied type material.

The large shell size (up to 50 mm in height) and distinctive yellow to orange body colour render *Pollicaria* very distinctive and easily recognizable, although some confusion might arise from the helicoid shape exhibited by juveniles. The fact that populations are often widely scattered and highly localized may account for their having been little studied and consequently poorly known (Crosse 1885, Kobelt 1902). Recently, karyotypic studies and preliminary allozyme analysis (Kongim et al. 2009, 2010, Panha unpub. data) have indicated that the species placed in synonymy by Kobelt (1902) and Pain (1974) should be recognized as distinct species.

Herein, we provide the first critical and comprehensive revision of *Pollicaria* based on a detailed morphological study of newly collected specimens and their comparison with type material.

## Materials and methods

Snails were collected and distributions recorded, mostly from limestone areas throughout Thailand, Laos, Vietnam and Peninsular Malaysia. Species identifications were made by comparison with type material, primarily at The Natural History Museum (London), Muséum National ďHistoire Naturelle (Paris), and University Museum of Zoology Cambridge (Cambridge). Living snails were photographed before examining the external and internal morphological characters. Adult shells were measured for height, diameter and whorl number. Features of the genitalia were examined for between 5 to 10 individuals of each species. Radulae were extracted and examined using a Scanning Electron Microscope (JEOL, JSM-5410 LV), and radular teeth shape and formulae were described.

**Anatomical abbreviation:** The following anatomical terminology used in this study was modified from Fischer (1885), Wenz (1938), Tielecke (1940) and Cox (1964): an, anus; at, atrium; bc, bursa copulatrix; cm, columellar muscle; ct, cephalic tentacles; dg, digestive gland; e, eye spots; ft, foot; h, head-portion of spermatophore; k, kidney; lc, lung cavity; me, mantle edge; op, operculum; ov, oviduct; p, penis; pcd, pericardium; pg, prostate gland; rt, rectum; sg, seminal groove; sr, seminal receptacle; st, stomach; t, tail-portion of spermatophore; ts, testis; ut, uterus; ven, ventricle.

**Institutional abbreviation: NHMUK**, The Natural History Museum, London; **CUMZ**, Chulalongkorn University, Museum of Zoology, Bangkok, Thailand; **MNHN**, Muséum National ďHistoire Naturelle, Paris; **RBINS**, Royal Belgian Institute of Natural Sciences, Brussels, Belgium; **SMF**, Forschungsinstitut und Naturmuseum Senckenberg, Frankfurt, a.m.; **UMZC**, University Museum of Zoology Cambridge, Cambridge; **ZMA**, Zoologische Museum of Amsterdam, Amsterdam; **ZMB**, Zoologisches Museum of Berlin, Berlin; **ZMMSU**, Zoological Museum of Mahasarakham University, Thailand.

## Results of morphological studies

**Figure 1.**
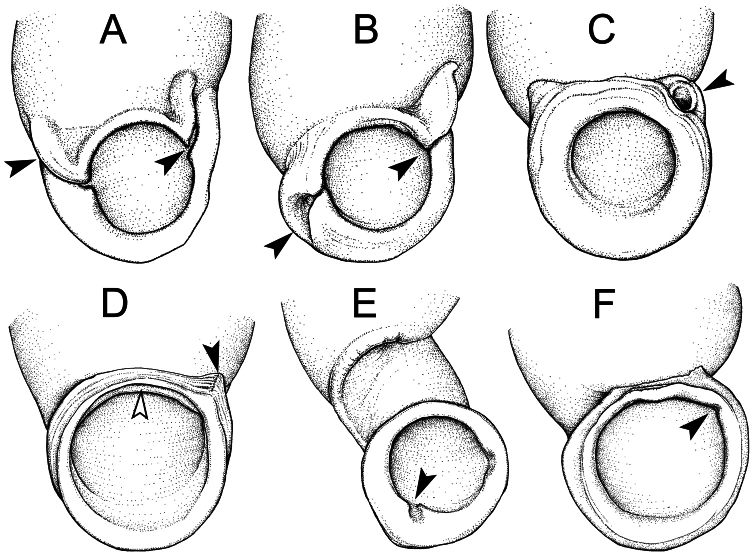
Breathing devices of six genera within the Pupinidae; black arrow indicates the position of breathing devices. **A** Anterior and posterior canals of *Pupina* **B** Anterior canal and twisted posterior canal of *Pupinella* **C** Complete posterior tube in *Raphaulus* **D** Shallow posterior angled groove of *Pollicaria*, white arrow indicates the parietal declining shoulder inside peristome **E** Anterior (peristomal) groove inside aperture of *Tortulosa* **F** Thin posterior groove inside aperture of *Schistoloma*.

**Shell characters:** *Pollicaria* Gould, 1856 is distinguished from other closely related genera such as *Pupina* Vignard, 1829, *Pupinella* Gray, 1850 and *Raphaulus* Pfeiffer, 1856 by having greater shell size, a breathing device in the form of a shallow posterior angled groove, and with or without a parietal declining shoulder inside the peristome (Fig. 1D). *Pupina* and *Pupinella* have anterior (columellar) and posterior (sutural) canals, with the columellar canal slightly twisted in *Pupinella* (Fig. 1A, B); *Raphaulus* has a complete posterior tube (Fig. 1C). *Pollicaria* differs from *Tortulosa* Gray, 1847 (Fig. 1E) and *Schistoloma* Kobelt, 1902 (Fig. 1F) by having a pupoid shell shape, larger shell size that lacks either an anterior (peristomal) groove (Fig. 1E) or posterior groove (Fig. 1F) respectively.

**Figure 2.**
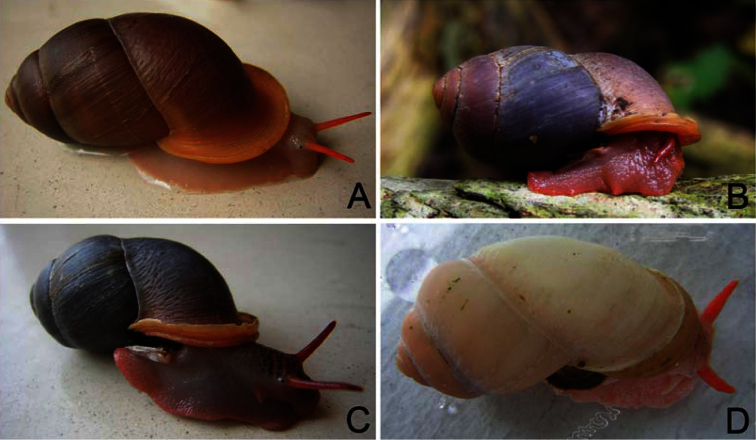
Living snails. **A** *Pollicaria myersii* from Pahom, Vang Vieng, Laos (CUMZ 1572; shell height about 40 mm) **B** *Pollicaria mouhoti mouhoti* from Tam Wungdang, Phitsanulok (CUMZ 1533; shell height about 35 mm) **C** *Pollicaria mouhoti monochroma* ssp. n. from the type locality (paratype CUMZ 1548; shell height about 30 mm) **D** *Pollicaria elephas* from Ipoh, Perak, Malaysia (CUMZ 1536; shell height about 45 mm).

**External features:** As recorded in the literature, *Pollicaria* was found to possess a yellow-orange to pale orange body, usually with dark orange cephalic tentacles (Fig. 2). Body colour variation within species appeared to be largely confined to patches of dark-brown or blackish spots spread across areas of the head and dorsal foot. Such variation may be present between different populations or can occur on different growth stages within populations.

**Figure 3.**
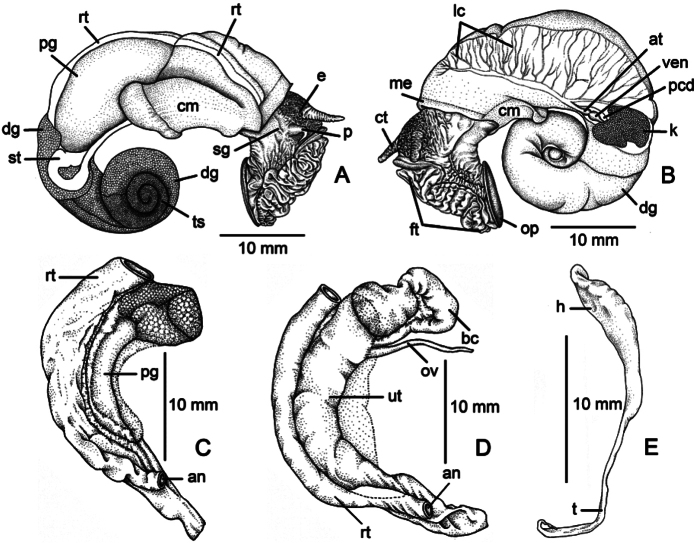
General anatomy, genitalia and spermatophore of *Pollicaria mouhoti mouhoti*. **A** Right side of snail showing male genital organ **B** Left side of snail showing pallial cavity and circulatory system **C** Male genital organ **D** Female genital organ **E** Spermatophore from uterus.

The foot (ft) is broad and short; cephalic tentacles (ct) long with dark eye spots (e) located at outer base; snout broad. Animal dioecious; genital groove present at right side running downwards from pallial gonoduct. Male with conical external penis (p) on the right side (penis usually broad and enlarged in breeding season) located below cephalic tentacles, and with seminal groove (sg) on penis (Fig. 3A); female with only genital groove on the right side disappear external penis. Operculum (op) attached to opercular lobe or disk posterior-dorsally on foot (Fig. 3B).

No external anatomical features were found to exhibit useful taxonomic characters.

**Internal anatomy:** The internal anatomical description of *Pollicaria mouhoti mouhoti* collected form Tam Wungdang, Nern Maprang, Phitsanulok, Thailand serves as being representative of the genus. Kidney (k) a brownish lobule, constricted-triangular in shape. Heart located on the left side of kidney; pericardium (pcd) thin, atrium (at) slightly larger than ventricle (ven). Lung cavity (lc) with reticulated vessels. Stomach (st) embedded in dark brown lobulated digestive gland (dg). Rectum (rt) large, attached to genital apparatus (prostate gland or uterus), and tapering anteriorly to anus (an), which opens close to mantle collar edge. Mantle edge (me) smooth and slightly thickened. Columellar muscle (cm) large, broad, thickened and whitish (Fig. 3A–D).

Testis (ts) with branched tubules, bright orange, occupying around 2-3 whorls from apex. Vas deferens thin and slender-straight tube attached to prostate gland at around two-third of its length proximal to external penis. Prostate gland (pg) large, long and slender, pale yellowish; proximally with genital opening. Seminal groove (sg) small, distinct and connected from genital opening on the right side of snail to external penis. External penis (pen) digitiform, short, located posteriorly, below cephalic tentacles (Fig. 3A, C).

Ovary bright orange multi-lobulated gland embedded in digestive gland. Pale yellow oviduct (ov) extends from ovary to uterus (ut) near the base of seminal receptacle. Bursa copulatrix (bc) cream to whitish long pouch that receives and digests the spermatophore case. Uterus (ut) large, curved pea-pod shape, posterior end rounded and anterior end tapering with genital opening (Fig. 3D).

Spermatophore tadpole shaped, about 20 mm long. Anterior portion or head of spermatophore (h) is a swollen pouch with thickened wall that is packed with sperm. Posterior portion or tail (t) tapering to slender tube is about half of the total length (Fig. 3E).

Both male and female genital organs of all species except *Pollicaria gravida* were examined and no distinguishing species-level taxonomic characters were found.

## Systematic account

### Family Pupinidae Pfeiffer, 1853

#### Genus Pollicaria

Gould, 1856

http://species-id.net/wiki/Pollicaria

Pollicaria Gould, 1856: 14. Gould 1862: 221. von Martens 1867: 67. Stoliczka 1871: 150. Kobelt 1902: 287. Wenz 1938: 475.
Hainesia Pfeiffer, 1856b: 120 (part.).
Hybocystis Benson, 1859: 90. Blanford 1864: 460. Crosse 1885: 180. Fischer 1885: 174.

##### Type species:

*Cyclostoma pollex* Gould, 1856: 14; by monotypy (see ICZN, 1999, Art. 68.3). The type species ‘*Cyclostoma pollex* Gould, 1856 [October]’ is currently recognized as a junior subjective synonym of *Megalomastoma gravidum* Benson, 1856 [March].

##### Note.

When describing his new species as *Cyclostoma pollex*, Gould (1856) simultaneously proposed the new generic name *Pollicaria* for this new nominal species. Gould also doubtfully included *Cyclostoma myersii* Haines, 1855 and *Cyclostoma chrysalis* Pfeiffer, 1852 in the *Pollicaria* Gould, 1856. Benson (1859) published a new generic name *Hybocystis* containing a single species from Burma *Megalomastoma gravidum* Benson, 1856. Although Benson (1860) noted that *Hybocystis* was a junior subjective synonym of *Pollicaria* Gould, 1856, the name *Pollicaria* was widely overlooked prior to Kobelt’s (1902) review of cyclophoroideans and both Wenz (1938) and Pain (1974) continued to mistakenly cite *Megalomastoma gravidum* Benson, 1856 as the type species. With only the doubtful inclusion of *Cyclostoma myersii* Haines, 1855 and *Cyclostoma chrysalis* Pfeiffer, 1852 in the original description of *Pollicaria*, the type species of *Pollicaria* was unequivocally fixed in the original publication by monotypy.

##### Diagnosis.

Shell pupoid, small to large (shell height 35–50 mm), thickened and solid. Shell smooth or malleated sculpture from almost white to pale yellow, reddish brown and nearly black; periostracum generally thick. Whorls 5–7, last whorl expanded, body whorl distorted when adult; sutures weakly impressed. Aperture rounded, shallow to absent posterior angled groove; peristome continuous and thickened; lip duplicated and reflexed; umbilicus narrow. Operculum multi-lamellar calcareous plate. Radula taenioglossate with seven teeth in each transverse row.

##### Keys to species and subspecies of the genus *Pollicaria* recognized in this study

**Table:** 

1	Peristome with declining shoulder inside peristome (Fig. 1A)	2
–	Peristome without declining shoulder inside peristome (Fig. 1B)	4
2	Shell small (height < 35 mm)	3
–	Shell large (height > 40 mm), shell ground colour brown to black, periostracum corneous	*Pollicaria rochebruni*
3	Shell pale yellow	*Pollicaria gravida*
–	Shell bright orange	*Pollicaria crossei*
4	Shell usually large (height > 40 mm)	5
–	Shell small (height < 35 mm) to medium (35 < height < 40 mm), with bright orange, purple to black	6
5	Shell dark orange to pale orange, lip duplicated, dorsal part of last whorl pitted	*Pollicaria elephas*
–	Shell elongate pupoid, brown to red, periostracum thick corneous, lip expanded, dorsal part of last whorl malleated	*Pollicaria myersii*
6	Spire and apex bright yellow to orange, shell medium (35 < height < 40 mm)	*Pollicaria mouhoti mouhoti*
–	Spire monochrome purple to black, shell small (height < 35 mm)	*Pollicaria mouhoti monochroma* ssp. n.

#### Pollicaria gravida

(Benson, 1856)

http://species-id.net/wiki/Pollicaria_gravida

**Figure 4.**
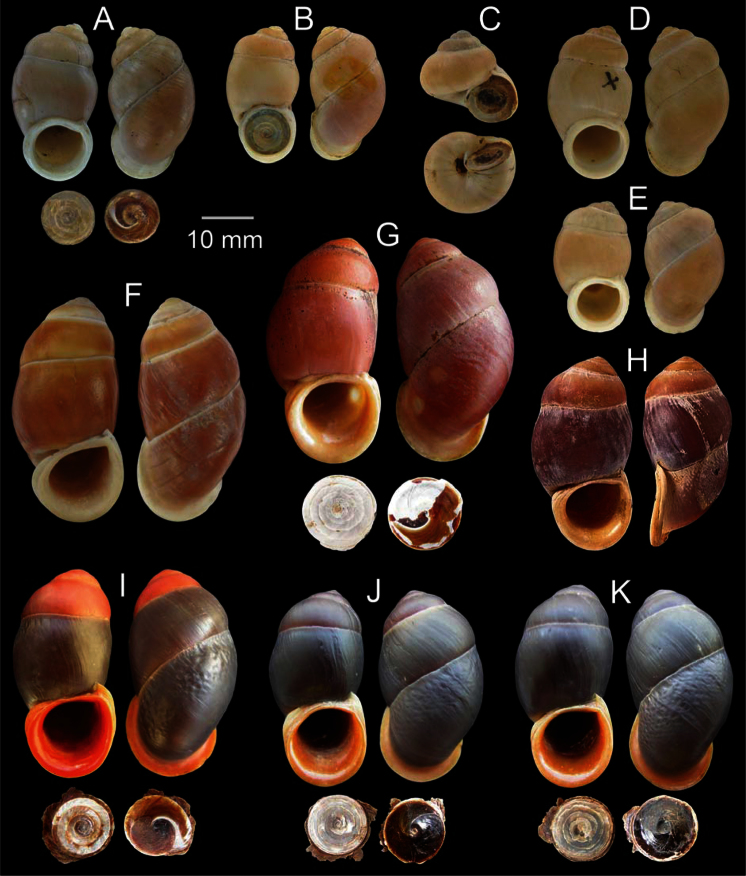
Shell morphology of *Pollicaria* spp. **A–E** *Pollicaria gravida* **A** lectotype UMZC I.102935.A **B** paralectotype UMZC I.102935.B-E **C** syntype of *Otopoma blennus* Benson, 1856 UMZC I.102930.A and **D, E** specimens from Farm Caves, Moulmein, Burma (NHMUK 88.124.863.4–5; specimen with ‘x’ was figured in Gude (1921), fig. 29) **F, G** *Pollicaria myersii* **F** form Siam (NHMUK 20090242; specimen figured in Pfeiffer, 1856a, pl. 19, figs 1, 2), and **G** specimen from Pahom, Vang Vieng, Laos (CUMZ 1572) **H, I** *Pollicaria mouhoti mouhoti* **H** lectotype (NHMUK 20130071/1), and **I** specimen from Tam Wungdang, Phitsanulok (CUMZ 1533) **J, K** *Pollicaria mouhoti monochroma* ssp. n. from the type locality, **J** holotype CUMZ 1577, and **K** paratype CUMZ 1548.

Fig. 4A–E Tables 1 2

Megalostoma gravidum Benson, 1856 [March]: 229. Type locality: Moulmein. Hanley and Theobald 1870: pl. 7, fig. 1.
Otopoma blennus Benson, 1856: 231. Type locality: Moulmein.
Cyclostoma pollex Gould, 1856 [October]: 14. Type locality: Tavoy, British Burma. Gould 1862: 221.
Hybocystis gravida —Benson, 1859: 91. Pfeiffer 1860: 123, 124, pl. 35, figs 1–4. Blanford 1864: 460. Crosse 1885: 187–190, pl. 11, fig. 2. Fischer 1885: 174.
Pollicaria gravida —Stoliczka, 1871: 150. Sowerby 1878: Pupinidae, pl. 8, species 68. Kobelt 1902: 289, 290, fig. 65. Gude 1921: 191, fig. 29. Wenz 1938: 475, fig. 1213. Pain 1974: 174, pl. 6 fig. 7.
Cyclostoma (Pollicaria) pollex —Johnson, 1964: 129.

##### Material examined.

Five shells in the type series of W.H. Benson, the specimen with similar shape, size and colour to the original description is designated here as the lectotype of *Megalomastoma gravidum* Benson, 1856 UMZC I.102935.A (height 32 mm, width 18 mm; Fig. 4A) and paralectotypes UMZC I.102935.B-E (4 shells, Fig. 4B); syntype of *Otopoma blennus* Benson, 1856 UMZC I.102930.A-B (2 shells, Fig. 4C).

Burma: NHMUK 79.9.1.5-6 (2 shells), Theobald colln. Acc. no. 1592 (2 shells), B.R. Lucus colln. Acc. no. 2351 (2 shells), Trechmann colln. Acc. no. 2176 (2 shells), 2 lots of E.R. Sykes collns. Acc. no. 1825 (1 shell and 2 shells); ZMA: R.v. Lennep colln. Acc. no. 1876 (1 shell); ZMB: Paetel colln. (1 shell), 2 lots of Dunker collns. (1 shell, 2 shells), Nevill colln. ZMB 20723 (2 shells). Farm Cave, Moulmein: NHMUK 88.124.863.4-5 (3 shells, Fig. 4D, E). Moulmein, Burma: NHMUK 71.9.23.193 (1 shell), 24.06.4.4 (2 shells), 1954.6.2.1231-1 (2 shells), H. Cuming colln. (4 shells), 2 lots of H.F./W.T. Blanford collns. Acc. no. 1944 (5 shells, 2 shells), T. Oldham colln. Acc. no. 1733 (2 shells); ZMA: Schepman colln. (1 shell). Unknown locality: NHMUK V.W. MacAndrew coll. (4 shells), H.E.J. Biggs colln. Acc. no. 2258 (2 shells), H. Cuming colln. (1 shell).

**Table 1.** Comparative morphological characters and karyotype among *Pollicaria* species recognized in this study.

**Characters**	***Pollicaria gravida***	***Pollicaria myersii***	***Pollicaria mouhoti***	***Pollicaria elephas***	***Pollicaria rochebruni***	***Pollicaria crossei***
Shell size (shell height)	small (height <35 mm)	large (height > 40 mm)	small to medium	large (height > 40 mm)	medium (35 < height > 40 mm)	small (height <35 mm)
Umbilicus	perforate	narrow	subumbilicate	narrow	narrow	narrow
Periostracum; shell colour	transparent; whitish to yellow	transparent; monochrome pale orange	transparent; monochrome black or with orange apex	transparent; yellow to orange	thicken corneous; reddish to orange	transparent; pale to deep orange
Sculpture on last whorl	absent	with thin wrinkle sculpture	with prominent wrinkle sculpture	with prominent wrinkle sculpture	absent	absent
Peristome shape; colour	rounded; as hell colour	slightly distorted; orange	slightly distorted; bright orange to reddish	rounded; as shell colour	rounded; as shell colour	rounded; as shell colour
Apertural groove	present	absent	absent	absent	present	present
Karyotype*	not available	4m+6sm+2st+ 1t	6m+4sm+2st+ 1t 7m+3sm+2st+ 1t	2m+6sm+2st+ 3t	3m+7sm+2st+ 1t	2m+8sm+2st+ 1t

* Data from Kongim et al. (2009, 2010); the chromosome morphology abbreviations: m, metacentric; sm, submetacentric; st, subtelocentric; t, telocentric.

**Table 2.** Shell size variation among *Pollicaria* species recognized in this study.

**Species, Locality and CUMZ nos.**	**Number of adult shell examined**	**Ranges, Mean ± SD in mm of:**			**Whorl ranges**
		**Shell Height**	**Shell Width**	**h/d Ratio**	
*Pollicaria gravida*					
UMZC and NHMUK collections	14	24.7–34.3 29.8 ± 2.54	14.6–19.0 17.4 ± 1.58	1.65–1.82 1.71 ± 0.12	5¾-6¼
*Pollicaria myersii*					
Pahom, Vang Vieng, Laos: 1520, 1572	10	37.8–50.6 43.4 ± 3.94	18.6–23.9 21.1 ± 1.68	1.97–2.16 2.06 ± 0.05	6¾-7
*Pollicaria mouhoti mouhoti*					
Namnao N. P., Phetchabun: 1538, 1574	7	36.2–41.5 37.6 ± 1.74	18.4–21.6 19.6 ± 0.96	1.85–1.99 1.92 ± 0.05	6½
Phu Kiew Wildlife Sanctuary, Chaiyaphum: 1551, 1528, 1529, 1571	65	33.6–44.1 37.7 ± 2.26	17.8–23.3 19.5 ± 1.20	1.86–2.14 1.94 ± 0.06	6–6¾
Tam Wungdang, Phitsanulok: 1533, 1537	40	33.4–40.8 36.7 ± 1.81	17.6–21.2 19.0 ± 0.83	1.86–2.05 1.93 ± 0.05	6–6½
Wat Pa-Mamuang, Phitsanulok: 1541	13	33.9–40.4 37.7 ± 2.12	18.4–20.2 19.6 ±0.78	1.82–2.04 1.92 ± 0.06	6–6½
*Pollicaria mouhoti monochroma* ssp. n.					
Phu Phalom, Loei: 1547	23	31.1–42.6 38.2 ± 2.52	17.9–21.4 19.4 ± 0.97	1.55–2.15 1.98 ± 0.11	6–6½
Tam Pha Bing, Loei: 1561, 1562	134	30.5–39.1 34.5 ± 2.09	16.4–20.6 18.4 ± 1.03	1.78–2.13 1.88 ± 0.05	5¾-6½
Tam Pha Singh, Loei: 1543	33	29.6–37.9 33.8 ± 2.41	15.8–19.5 17.6 ± 1.03	1.71–2.00 1.92 ± 0.06	5¾-6¼
Wat Tam Pha Poo, Loei: 1545	56	30.2–36.7 32.5 ± 1.37	16.1–19.3 17.2 ± 0.64	1.76–2.00 1.89 ± 0.05	5¾-6
*Pollicaria elephas*					
Ampang Baru, Ipoh, Perak, Malaysia: 1535	51	36.4–51.1 43.9 ± 3.12	19.4–24.5 21.9 ± 1.31	1.85–2.12 2.01 ± 0.06	6–6¾
Gunung Kenting, Ipoh, Perak, Malaysia: 1534, 1536, 1596	182	36.4–49.9, 42.3 ± 2.34	11.1–25.6 21.9 ± 1.64	1.78–3.79 1.4 ± 0.15	6–6¾
*Pollicaria rochebruni*					
Cuc Phuong N. P., Vietnam: 1521, 1532	8	32.6–42.3 40.1 ± 2.26	18.1–22.9 20.6 ± 1.66	1.80–2.09 1.95 ± 0.11	6¼
Phuong Nga N. P., Vietnam: 1523, 1539, 1552	5	37.8–45.0 40.8 ± 2.74	20.0–21.8 20.7 ± 0.71	1.89–2.07 1.97 ± 0.07	6¼-6¾
*Pollicaria crossei*					
Cuc Phuong N. P., Vietnam: 1521, 1522, 1588	10	32.6–38.0 35.3 ± 1.74	17.6–18.9 18.9 ± 1.01	1.82–1.93 1.87 ± 0.06	6¼
Hulien, Vietnam: 1590	5	32.7–36.2 34.9 ± 1.43	18.0–19.8 18.7 ± 0.07	1.82–1.93 1.87 ± 0.05	6¼

##### Description. 

**Shell.** Shell small for *Pollicaria*, pupoid, pale orange, yellow to white. Periostracum thin and transparent; shell surface smooth. Whorls 5–6; sutures moderately impressed; apex slightly inclined to right; spire short. Last whorl large about two-thirds of shell height, distorted and flattened in front. Aperture rounded with a shallow posterior angled groove. Peristome continuous, with distinct parietal declining shoulder internally. Lip thickened, little expanded, and margin moderately duplicated; umbilicus narrow. Operculum thick, calcareous, multispiral.

##### Distribution.

Accepted records are confined to Burma: Moulmein, Damontha, Tavoy and Tenasserim (Benson 1856, 1859, Stoliczka 1871, Crosse 1885, Kobelt 1902, Gude 1921, Pain 1974). Records from Northern Vietnam of *Pollicaria crossei* and *Pollicaria rochebruni* are considered to be distinct species.

##### Remarks.

**Figure 5.**
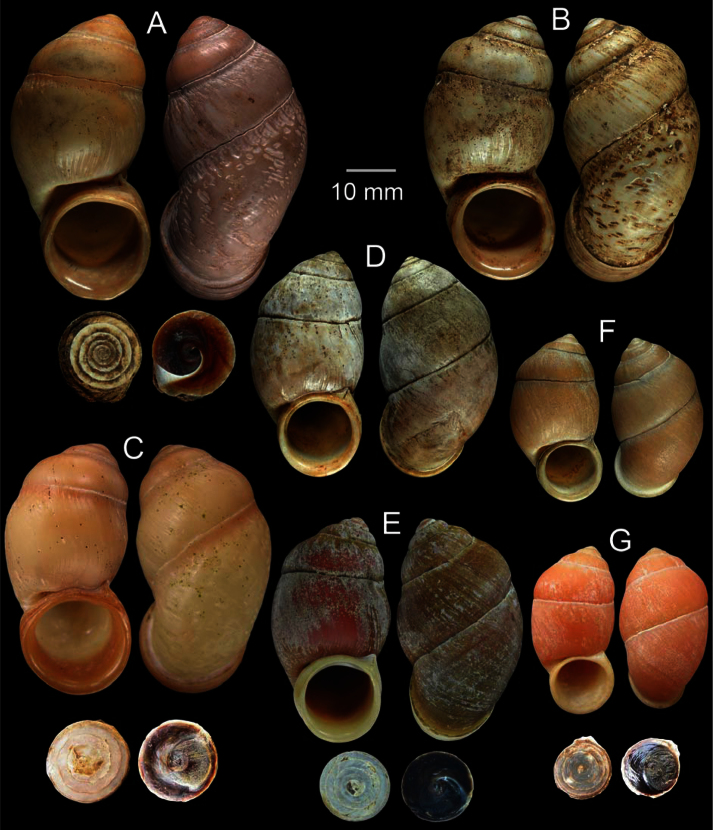
Shell morphology of *Pollicaria* spp. **A–C** *Pollicaria elephas* **A** lectotype of *Pollicaria elephas* (MNHN 21309) **B** lectotype of *Hybocystis jousseaumei* Morgan, 1885 (MNHN 21308), and **C** specimen from Gunung Kenting, Ipoh, Perak, Malaysia (CUMZ 1536) **D, E** *Pollicaria rochebruni* **D** lectotype (MNHN 21305), and **E** specimen from Phuong Nga National Park, Vietnam (CUMZ 1568) **F, G** *Pollicaria crossei*, **F** lectotype (MNHN 21304), and **G** specimen from Cuc Phuong National Park, Vietnam (CUMZ 1588).

*Otopoma blennus* Benson, 1856 and *Cyclostoma pollex* Gould, 1856 have long been considered as junior synonyms of *Pollicaria gravida* and this classification has been followed by a number of authors (Hanley and Theobald 1870, Sowerby 1878, Crosse 1885, Kobelt 1902, Pain 1974). Subsequently, *Pollicaria crossei* and *Pollicaria rochebruni* from Vietnam were also placed into synonymy with this species (see Pain 1974). However, examination of the type specimens of these three species (Figs 4A, B; 5D, F) demonstrated that *Pollicaria gravida* could be distinguished from *Pollicaria crossei* and *Pollicaria rochebruni* by having a whitish to yellowish shell colour with swollen whorls, impressed sutures and with the last whorl flattened ventrally (Table 1). Furthermore, *Pollicaria gravida* is mainly restricted to the western edge of the *Pollicaria* distribution in Tavoy and Tenasserim of Burma, and does not overlap with the two Vietnamese species in the east (Pain 1974). Unfortunately, none of the live specimens of *Pollicaria gravida* were examined cytogenetically for additional discrimination of these three species.

#### Pollicaria myersii

(Haines, 1855)

http://species-id.net/wiki/Pollicaria_myersii

Figs 2A 4F, G 6A Tables 1 2

Cyclostoma (Megalostoma) myersii Haines, 1855: 157, pl. 5, fig. 9–11. Type locality: Siam.
Megalostoma myersi —Pfeiffer, 1856a: 67, pl. 19, figs 1, 2.
Megalostoma (Hainesia) myersi —Pfeiffer, 1856b: 120.
Megalostoma myersii —von Martens, 1860: 11.
Pollicaria myersi —von Martens, 1867: 67. Sowerby, 1878: Pupinidae, pl. 8, species 69. Kobelt, 1902: 290.
Hybocystis myersi —Crosse, 1885: 191–193, pl. 11, fig. 4.
Pollicaria myersii —Habe, 1964: 114, pl. 2, fig. 13. Pain, 1974: 175, 176, pl. 6, figs 2, 5.

##### Material examined.

Siam: NHMUK 20090242 (Fig. 4F). Pahom, Vang Vieng, Laos: CUMZ 1531, 1572 (Fig. 4G), 1591; ZMMSU 0009.

##### Description. 

**Shell:** Shell large, reddish brown to light orange. Periostracum thin, corneous; shell surface usually with fine malleations on upper half of last whorl. Aperture almost circular with a shallow posterior angled groove. Peristome yellow, parietal declining shoulder absent. Lip thickened, broadly expanded, reflexed, with concentric margin.

**Radula:** Radular teeth arranged in v-shaped rows, each transverse row with 7 teeth (2-1-1-1-2). Central tooth with well developed central cusp and one smaller lateral cusp on each side; central cusp large, elongate with pointed tip. Lateral teeth with 2 cusps, outer cusp largest and elongate shape with pointed tip, and with relatively small pointed tip of inner lateral cusps. Inner and outer marginal teeth with 2 cusps; central cusp large, flanked by small inner lateral cusps.

##### Distribution:

The type locality of this species was given as the broad location of “Siam” (see Haines 1855). Subsequently, *Pollicaria mouhoti* was synonymised with *Pollicaria myersii* (von Martens 1867, Pain 1974) thus expanding the distribution of *Pollicaria myersii* beyond its historical range. However, in this study the distribution of the species is restricted to limestone areas of Vientiane to Luang Prabang, Laos, and probably the northern part of Thailand.

##### Remarks:

The syntype AMNH 43629 could not be traced (Siddal and Watson, personal communication). Due to the proximity of the geographic distributions and similarity in shell morphology of the two species, *Pollicaria mouhoti* have long been considered a junior synonym of *Pollicaria myersii* (see Pain 1974). However, *Pollicaria myersii* can be distinguished from *Pollicaria mouhoti* by an elongated purple to pale orange shell with thin periostracum, rounded aperture and very fine wrinkles on the dorsal part of the last whorl (Table 1, Fig. 4F, K). *Pollicaria myersii* differs from *Pollicaria gravida*, *Pollicaria rochebruni* and *Pollicaria crossei* by having a larger shell, no apertural groove and noticeable wrinkles on last whorl (Tables 1, 2).

**Figure 6.**
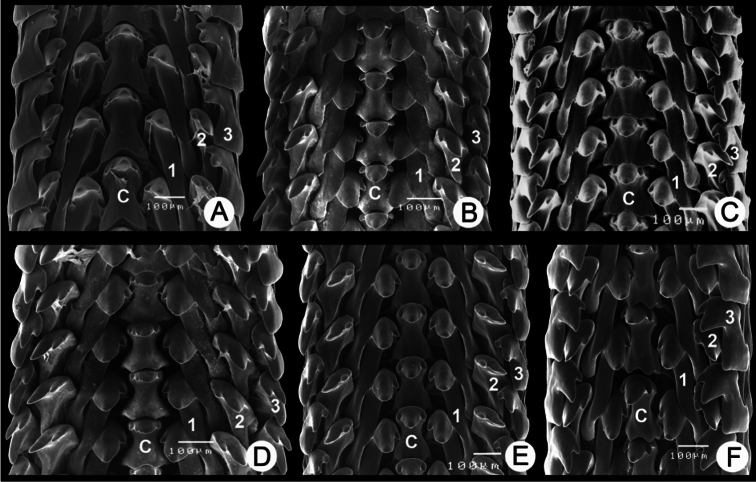
Radular morphology of *Pollicaria* spp. **A** *Pollicaria myersii* from Pahom, Vang Vieng, Laos (CUMZ 1572) **B** *Pollicaria mouhoti mouhoti* from Tam Wungdang, Phitsanulok (CUMZ 1533) **C** *Pollicaria mouhoti monochroma* ssp. n. from the type locality (paratype CUMZ 1548) **D** *Pollicaria elephas* from Gunung Kenting, Ipoh, Perak, Malaysia (CUMZ 1536) **E** *Pollicaria rochebruni* from Phuong Nga National Park, Vietnam (CUMZ 1568) **F** *Pollicaria crossei* from Cuc Phuong National Park, Ninh Binh Province, Vietnam (CUMZ 1588). Numbers indicated order of lateral and marginal teeth. Central tooth indicated by ‘**C**’.

#### Pollicaria mouhoti

(Pfeiffer, 1862)

http://species-id.net/wiki/Pollicaria_mouhoti

Hybocystis mouhoti Pfeiffer, 1862: 276, pl. 36, fig. 13. Type locality: Laos Mountain, Cambodia. Pfeiffer 1863: 227, 228, pl. 59, figs 5–8. Crosse 1885: 190,191, pl. 11, fig. 3.
Megalostoma (Hybocystis) mouhoti —von Martens, 1867: 67.
Pollicaria mouhoti —Sowerby, 1878: Pupinidae, pl. 8, species 67. Kobelt 1902: 290.

##### Diagnosis.

Shell small to large, pupoid, solid; monochrome purple to black, sometimes with yellowish to bright orange spire. Periostracum thin; shell surface with distinct malleations on upper half of last whorl. Whorls 5-6; sutures moderately impressed; apex obtuse. Last whorl large about two-thirds of shell height, distorted and flattened in front. Aperture almost circular, shallow posterior angled groove present. Peristome and inside aperture orange to red; parietal declining shoulder absent. Lip thickened, expanded, reflexed, margin slightly duplicated; umbilicus narrow. Operculum calcareous concentric.

##### Distribution.

The type locality of *Pollicaria mouhoti* was given as Laos Mountain, Cambodia. However, subsequent records of this species were from Thailand, Laos and Cambodia (Pfeiffer 1862, Crosse 1885, Kobelt 1902, Solem 1966).

##### Remarks.

von Martens (1867) and Pain (1974) synonymised this species with *Pollicaria myersii* and stated that all *Pollicaria* specimens collected from Thailand should be regarded as this species. However, examination of the type specimens of *Pollicaria mouhoti* (Fig. 4H) showed that it was clearly distinct from *Pollicaria myersii* in shell shape, sculpture and colour pattern. The major distinguishing shell characters of *Pollicaria mouhoti* are the smaller shell size, purplish shell colour, bright orange spire, expanded bright orange to red apertural lip and bold wrinkles on the dorsal side of last whorl (Tables 1, 2). In addition, the chromosome analysis shows a clear difference in karyotype patterns between these two species (Kongim et al. 2009, 2010). Hence, *Pollicaria mouhoti* is removed from the synonymy of *Pollicaria myersii* and reinstated as a distinct species.

#### Pollicaria mouhoti mouhoti

(Pfeiffer, 1862)

http://species-id.net/wiki/Pollicaria_mouhoti_mouhoti

Figs 2B 3A_E 4H, I 6B Tables 1 2

##### Material examined.

Three syntype shells in H. Cuming collection, the figures and labels with type specimen are designated here as the lectotype of *Hybocystis mouhoti* Pfeiffer, 1862 NHMUK 20130071/1 (height 34.2 mm, width 18.1 mm; Fig. 4H) and paralectotype NHMUK 20130071/2-3 (2 shells). Cambodia: ZMA Wright colln. (2 shells), R.v. Lennep colln. (1 shell). Laos Mountain: ZMB Paetel colln. (1 shell). Phu Kradung, Loei: CUMZ 1586. Namnao National Park, Phetchabun: CUMZ 1574, 1538; ZMMSU 0002. Tam Yai Namnao, Phetchabun: CUMZ 1559. Phu Phaman, Khon Kaen: ZMMSU 0012. Phu Kiew Wildlife Sanctuary, Nongbuadang, Chaiyaphum: CUMZ 1528, 1529, 1551, 1571, 1576, 1582, 1585; ZMMSU 0003, 0020-4, 0027, 0029. Phu Phachit, Chaiyaphum: ZMMSU 0013. Tam Tao, Nernmaprang, Phitsanulok: CUMZ 1558. Tam Wungdang, Nernmaprang, Phitsanulok: CUMZ 1533 (Fig. 4I), 1537, 1544, 1554, 1575. Wat Pa Mamuang, Nernmaprang, Phitsanulok: CUMZ 1541; ZMMSU 0015. Wat Thepitakpunnaram, Pakchong, Nakhon Ratchasima: CUMZ 1583. Tam Pu Loop, Phuphaman, Khon Kaen: CUMZ 1526. Namprom Dam, Khon Kaen: CUMZ 1584.

##### Description. 

**Shell:** This nominotypical subspecies is characterized by the large shell size (Table 2). Shell with last whorl and penultimate whorl purple to black; first to third whorls distinct yellow to bright orange. Lip expanded, red to orange.

**Radula:** Taenioglossate radula, teeth arrangement with central, lateral and marginal teeth shape similar to *Pollicaria myersii*. Differences include a central tooth with well developed central cusp and lateral cusp on each side; lateral teeth triangular in shape with a pointed tip; inner marginal teeth composed of 3 cusps; central cusp flanked with small inner and outer lateral cusps.

##### Distribution.

This subspecies occupies the southern limit of the species’ range in Cambodia and several localities in Loei, Phitsanulok, Chaiyaphum, Khon Kaen, Phetchabun Nakhon Ratchasima and Saraburi Provinces in Thailand.

##### Remarks.

The characters distinguishing this nominotypical subspecies from *Pollicaria myersii* are the smaller shell size and mainly purple coloured shell with whorls 2-3 pale to bright orange and bright orange to red lip (Tables 1, 2), and a distinct karyotype pattern (Kongim et al. 2009, 2010).

#### Pollicaria mouhoti monochroma

Kongim & Panha ssp. n.

http://species-id.net/wiki/Pollicaria_mouhoti_monochroma

Figs 2C 4J, K 6C Tables 1 2

##### Type material.

Holotype: CUMZ 1577 (Fig. 4J; height 34.5 mm, width 18.4 mm, 6½ whorls) from the type locality, paratypes CUMZ 1548 (Fig. 4K; 9 shells), 1561 (82 shells), 1562 (85 shells); NHMUK 20130073 (5 shells); MNHN IM-2012-2103; SMF341492 (5 shells).

##### Type locality.

Limestone outcrop with dry forest at Wat Tam Pha Bing, Wungsapoong District, Loei Province, Thailand (17°14'1.3"N, 101°44'3.5"E).

##### Other material examined.

Phakeng-Phanang, Loei: ZMMSU 0025, 0026. Phu Luang Wildlife Sanctuary, Loei: CUMZ 1524. Phu Phalom, Muang, Loei: CUMZ 1547, 1560, 1565, 1567, 1580. Phu Phasamyod, Loei: ZMMSU 0011. Tam Erawan, Wungsapoong, Loei: CUMZ 1555, 1579. Tam Pha Bing, Wungsapoong, Loei: CUMZ 1548, 1561, 1562, 1577, ZMMSU 0001, 0004, 0006, 0017, 0028. Tam Pha Singh, Wungsapoong, Loei: CUMZ 1543, 1546. Wat Po Thi-sat, Nonghin, Loei: CUMZ 1557. Wat Tam Kuhawari, Nonghin, Loei: CUMZ 1540, 1549. Wat Tam Pha Mak-ho, Wungsapoong, Loei: CUMZ 1530, 1542. Wat Tam Pha Poo, Loei: CUMZ 1545, 1550. Wat Tam Piya, Loei: CUMZ 1527. Khao Wungpha, Nawung, Nongbua Lumphoo: CUMZ 1563, 1564. Nawung, Nongbua Lumphoo: CUMZ 1581. Tam Suwankuha, Nongbua Lumphoo: ZMMSU 0007.

##### Etymology.

From the Greek *monos* = one or single, and *chroma* = color of the skin; referring to the characteristic uniform dark brown to blackish spire color of the shell.

##### Description. 

**Shell:** Shell relatively small, pupoid, monochrome purple to black. Periostracum thin and transparent. Whorls 5-6; sutures moderately impressed; apex obtuse; spire short. Last whorl large about two-thirds of shell height, flattened in front. Shell surface rough with malleations on upper half of last whorl. Aperture almost circular, shallow posterior angled groove present; peristome continuous, yellow to pale orange. Lip thickened, broadly expanded; umbilicus narrow. Operculum thick, calcareous, concentric, exterior little concave.

**Radula:** Taenioglossate radula, teeth arrangement with central, lateral and marginal teeth shape similar to the nominotypical subspecies.

##### Distribution.

*Pollicaria mouhoti monochroma* ssp. n. is restricted to the northern limit of the species’ distribution in Loei, Phetchabun, Chaiyaphum and Nongbua Lumphoo Provinces.

##### Remark.

*Pollicaria mouhoti monochroma* ssp. n. can be distinguished from the nominotypical subspecies by having a much smaller, entirely black to purple shell (Tables 1, 2) and a distinct karyotype pattern (see Kongim et al. 2009, 2010). The shell size and shape of this subspecies are similar to that of *Pollicaria gravida* and *Pollicaria crossei*, but the purple shell is a distinguishing characteristic.

Shell character variations can be observed in the Phu Pha Lom, Loei Province population. These individuals exhibit a relatively larger shell than the typical populations (Table 2), however, the monochrome black shell and similar karyotype pattern indicate that they belong to this subspecies (Kongim et al. 2009).

#### Pollicaria elephas

(Morgan, 1885)

http://species-id.net/wiki/Pollicaria_elephas

Figs 2D 5A–C 6D Tables 1 2

Hybocystis elephas Morgan, 1885b: 70. Type locality: Perak. Morgan 1885a: 404, 405, pl. 7, fig. 1. Crosse 1885: 183–186, pl. 11, fig. 1. Fischer 1885: 174. Möllendorff 1886: 314. Möllendorff 1891: 346. Kobelt and Möllendorff 1899: 137.
Hybocystis jousseaumei Morgan, 1885b: 70. Type locality: Kinta, Perak. Morgan 1885a: 405, 406, pl. 7, fig. 2. Crosse 1885: 184.
Pollicaria elephas —Kobelt, 1902: 289. Laidlaw 1928: 33. van Benthem Jutting 1960: 12. Pain 1974: 176, pl. 6, fig. 1, 3. Abbott 1989: 46, 1 figure. Chan 1997: 11, fig. 1–2.

##### Material examined.

Five lots with 13 specimens of syntype deposited in MNHN, the specimen figured in the original publication is designated as the lectotype of *Hybocystis elephas* Morgan, 1885 MNHN 21309 (Fig. 5A), paralectotype MNHN 21310 (5 shells), 21311 (2 shells), 21312 (3 shells), 21313 (2 shells), RBINS 525391 (1 shell). Single syntype specimen is designated as the lectotype of *Hybocystis jousseaumei* Morgan, 1885 MNHN 21308 (Fig. 5B). Ipoh, Perak, Malaysia: ZMA E.A. Meene colln. Acc. no. 1982 (1 shell). Near bridge over river, road Ipoh to Tanjong Rambutan, Perak, Malaysia: ZMA J. Drijver colln. (5 shells). Perak, Malaysia: ZMB 75821 (2 shells), 38044 (1 shell), M. Schulz colln. 1216 (3 shells, smallest shell excluded). Bukit Chintamani, Selangor, Malaysia: CUMZ 1534. Gunung Kenting, Ampang Baru, Ipoh, Perak, Malaysia: CUMZ 1535, 1536 (Fig. 5C), 1566, 1570.

##### Description. 

**Shell:** Shell large, elongate pupoid uniform yellow to orange. Periostracum thin, corneous; shell surface with fine growth lines and last whorl with distinctly strong pitting dorsally. Whorls 6-7 whorls; sutures impressed; apex obtuse. Last whorl large about two-third of shell height, flattened in front. Aperture rounded, with shallow to deep posterior angle groove. Peristome continuous, little elevated, yellow to orange, internal parietal declining shoulder absent. Lip thickened, duplicated, and with distinct growth ridges; umbilicus narrow. Operculum thick, calcareous, concentric.

**Radula:** Taenioglossate radula, teeth arrangement with central, lateral and marginal teeth shape similar to *Pollicaria myersii*. Minor differences are the well-developed central cusp with one to three small lateral cusps of the central tooth, and the slightly elongate and slender central cusp of the inner marginal teeth.

##### Distribution.

This species has a restricted distribution and is known only from limestone outcrops in Perak, Peninsular Malaysia (Morgan 1885a, b). Material collected for this study was from Kinta valley, Perak, and the southern part of the species’ historical range in Bukit Chintamani, Selangor, Peninsular Malaysia is considered to be this locally endemic species.

##### Remarks.

The locally endemic *Pollicaria elephas* is confined to a few limestone outcrops in Peninsular Malaysia and shows several unique shell characters that separate it from its congeners. The major distinguishing characters of this species are the very large, monochrome yellowish to pale orange shell with the last whorl distorted ventrally and sculptured with scattered, deep pits dorsally; and rounded and thickened aperture. (Table 2, Fig. 5A–C).

Morgan (1885a, b) proposed two nominal species of *Pollicaria* from Perak, which differed mainly by the shell size (larger shell *Hybocystis elephas* and smaller shell *Hybocystis jousseaumei*). In the first revision of this genus, Crosse (1885) assumed that they were the same species and recognized only *Pollicaria elephas*. Thereafter *Pollicaria jousseaumei* was recognized as a synonym of *Pollicaria elephas* (Kobelt 1902, Pain 1974). Examination of the type specimens (Fig. 5A, B) confirmed *Pollicaria jousseaumei* as junior synonym of *Pollicaria elephas*. Moreover, the recent land snail survey in Perak, Peninsular Malaysia recorded both large and small shell forms of the species from the same localities.

#### Pollicaria rochebruni

(Mabille, 1887)

http://species-id.net/wiki/Pollicaria_rochebruni

Figs 5D, E 6E Tables 1 2

Hybocystis rochebruni Mabille, 1887a: 12. Type locality: Tonkin. Mabille 1887b: 138, 139, pl. 2, figs 12, 13.
Pollicaria rochebruni —Kobelt, 1902: 290.

##### Material examined.

Four specimens of the syntype deposited in MNHN, the figured specimen in original publication is designated here as the lectotype of *Hybocystis rochebruni* Mabille, 1887 MNHN 21305 (Fig. 5D) and other specimens as paralectotype MNHN 25855. Bac Ma National Park, Vietnam: CUMZ 1556. Hulien Nature reserve, Vietnam: CUMZ 1594. Khe Sen, Danang, Vietnam: CUMZ 1589. Phuong Nga National Park, Quang Binh, Vietnam: CUMZ 1523, 1539, 1552, 1568 (Fig. 5E). Cuc Phuong National Park, Ninh Binh, Vietnam: CUMZ 1532, 1568, 1573, 1587.

##### Description. 

**Shell:** Shell medium-sized, pupoid, red-brown. Periostracum thick, corneous; shell surface smooth. Whorls 5-6; sutures moderately impressed; apex obtuse; spire short. Last whorl large about two-thirds of shell height, distorted and flattened in front, ventrally rounded. Aperture rounded, shallow to absent posterior angled groove present. Peristome continuous, with thin parietal declining shoulder internally. Lip thickened, little expanded, margin moderately duplicated with thin growth ridges; umbilicus narrow. Operculum concentric, thick, calcareous, multi-spiral plate.

**Radula:** Taenioglossate radula, teeth arrangement with marginal teeth shape similar to *Pollicaria myersii*. Major differences are in the central teeth which have multiple cusps: the central cusp relatively short and small, flanked by 1-3 tapered lateral cusps; and inner marginal teeth with 3 cusps: the central cusp large with a convex tip, flanked by small and pointed inner cusps, the outer lateral cusp very small to nearly wanting.

##### Distribution.

The previous records of this species were from Tonkin (Mabille 1887a, b); Babe National Park, Bac Kan, Vietnam (Yamazaki et al. 2007)

##### Remarks.

Based on the similarity in shell morphology, Pain (1974) placed *Pollicaria rochebruni* into the synonymy of *Pollicaria gravida*. However, examination of the type specimens of *Pollicaria rochebruni* indicate that it is a distinct species (see also *Pollicaria gravida*). *Pollicaria rochebruni* can be distinguished from the latter species by having a larger red-brown to purple-black shell with flattened whorls and shallow sutures, while *Pollicaria gravida* usually has smaller pale orange shell with convex whorls and impressed sutures (Tables 1, 2). *Pollicaria rochebruni* differs from the sympatric *Pollicaria crossei* in both shell size and colour (Tables 1, 2, Fig. 5F) as well as having a distinct karyotype pattern (see Kongim et al. 2010).

#### Pollicaria crossei

(Dautzenberg and ďHamonville, 1887)

http://species-id.net/wiki/Pollicaria_crossei

Figs 5F, G 6F Tables 1 2

Hybocystis crossei Dautzenberg and ďHamonville, 1887: 220, pl. 8, fig. 4. Type locality: Than Moi, Tonkin. Kobelt and Möllendorff 1899: 137. Kobelt 1902: 290.
Pollicaria crossei —Kobelt, 1902: 290.

##### Material examined.

Single specimens of the syntype deposited in MNHN, the figured specimen in original publication is designated here as the lectotype of *Hybocystis crossei* Dautzenberg and ďHamonville, 1887 MNHN 21304 (Fig. 5F), and paralectotype RBINS 525390 (3 shells; the biggest one excluded). Cuc Phuong National Park, Ninh Binh, Vietnam: CUMZ 1521, 1522, 1588 (Fig. 5G), 1593. Hulien Nature reserve, Vietnam: CUMZ 1590.

##### Description. 

**Shell:** Shell small, pupoid, bright orange. Periostracum thin, corneous; shell surface smooth. Whorls 5-6; sutures moderately impressed; apex obtuse; spire short. Last whorl large about two-thirds of shell height, distorted and flattened in front, ventrally rounded. Aperture rounded, with shallow to absent posterior angled groove. Peristome continuous, with thin parietal declining shoulder internally. Lip thickened, little expanded and duplicated; umbilicus narrow. Operculum thick, calcareous, concentric.

**Radula:** Taenioglossate radula, teeth arrangement with central, lateral and marginal teeth similar in shape to *Pollicaria myersii*.

##### Distribution.

The previous records of *Pollicaria crossei* was from Than-Moi, Tonkin and Cuc Phuong National Park, Ninh Binh, Vietnam (Dautzenberg and ďHamonville 1887, Vermeulen and Maassen 2003).

##### Remarks.

*Pollicaria crossei* has long been recognized as a subjective synonym of either *Pollicaria rochebruni* or *Pollicaria gravida* (Kobelt 1902, Pain 1974). However, the relatively smaller bright orange shell with thick, brown periostracum of *Pollicaria crossei* are a combination of characters that distinguish it from *Pollicaria rochebruni*. The bright orange shell with flattened whorls and shallow sutures distinguish it from *Pollicaria gravida* (Table 1, Fig. 3A). Moreover, the karyotypic study of the smaller shell form of *Pollicaria gravida* sensu lato indicated a distinct species recognized as *Pollicaria crossei* (see Kongim et al. 2010).

## Supplementary Material

XML Treatment for Pollicaria

XML Treatment for Pollicaria gravida

XML Treatment for Pollicaria myersii

XML Treatment for Pollicaria mouhoti

XML Treatment for Pollicaria mouhoti mouhoti

XML Treatment for Pollicaria mouhoti monochroma

XML Treatment for Pollicaria elephas

XML Treatment for Pollicaria rochebruni

XML Treatment for Pollicaria crossei
